# Clinical Forms and *GRIN2A* Genotype of Severe End of Epileptic-Aphasia Spectrum Disorder

**DOI:** 10.3389/fped.2020.574803

**Published:** 2020-11-06

**Authors:** Xiao Li, Ling-Ling Xie, Wei Han, Si-Qi Hong, Jian-Nan Ma, Juan Wang, Li Jiang

**Affiliations:** ^1^Department of Neurology, Children's Hospital of Chongqing Medical University, Chongqing, China; ^2^Ministry of Education Key Laboratory of Child Development and Disorders, Chongqing, China; ^3^National Clinical Research Center for Child Health and Disorders, Chongqing, China; ^4^China International Science and Technology Cooperation base of Child Development and Critical Disorders, Chongqing, China; ^5^Chongqing Key Laboratory of Pediatrics, Chongqing, China

**Keywords:** Landau-Kleffner syndrome (LKS), epileptic encephalopathy with continuous spike-and-wave during sleep (CSWS), atypical benign partial epilepsy (ABPE), genotype-phenotype relationship, epileptic-aphasia spectrum disorder

## Abstract

**Objective:** This study aims to analyze the electroclinical characteristics and gene test results of children on the severe end of the epilepsy aphasia spectrum (EAS) and also the correlation of EAS-related GRIN2A genes to explore the genotype-phenotype relationships, as well as potential pathogenic mechanism of EAS.

**Methods:** A retrospective study was conducted on the participants diagnosed with Landau-Kleffner syndrome (LKS), epileptic encephalopathy with continuous spike-and-wave during sleep (CSWS), and atypical benign partial epilepsy (ABPE) at the Children's Hospital of Chongqing Medical University from January 2013 to June 2019. Whole-exome sequencing was performed in six patients, and epileptic panel was carried out in two. In addition, we reviewed all the published literatures reporting EAS patients with pathogenic variants until June 2019 and conducted Gene Ontology (GO) analysis, as well as protein-protein interaction (PPI) network.

**Results:** The mean age at seizure onset was 55.4 ± 27.0 months. The baseline severity of the spike-wave index (SWI) was not significantly correlated with intellectual disability (ID) level. Two pathogenic *de novo GRIN2A* null variants were identified in patients with ABPE who had less severe ID, despite the electrical status epilepticus during slow-wave sleep (ESES). By literature reviewing, 18 *GRIN2A* missense mutations and 11 *GRIN2A* truncating mutations which lead to *N*-methyl-d-aspartate receptors' loss of function has been reported. Of these mutations, 9 (31.0%) are situated in amino (N)-terminal domain, 6 (20.7%) in linger-binding domain S1, and 10 (34.5%) in linger-binding domain S2. EAS-related genes were enriched in the biological process of chemical synaptic transmission and vocalization (FDR, <0.01). The hub protein in PPI network is GluN2A, which might affect language function via foxp2-srpx2/uPAR signal network.

**Conclusion:** Our data suggested that when children suspected with benign epilepsy of children with centrotemporal spikes (BECTs) have early-onset age, changed seizure semiology, and deterioration of behavior/cognition/motor function, neurologists should be alert of the appearance of ESES. The neuropsychological deterioration in children with EAS might not only be completely affected by electric discharge severity but also genetic etiology. Our finding also enforced the current genotype-phenotype relationship theory about EAS. For EAS children, GRIN2A-FOXP2-SRPX2/uPAR signal network might contribute to the mechanism of their language deficit.

## Introduction

Benign epilepsy of children with centrotemporal spikes (BECTs) is the most common idiopathic focal epileptic syndrome in childhood, accounting for 15–25% of childhood-onset epilepsy. It is characterized by focal sensorimotor attack and typical EEG signal showing high amplitude spike wave in central temporal area, followed by a slow wave. The prognosis of BECTs was considered to be good, mostly relieved before puberty. However, with the broader utilization of neuropsychological evaluations, some cognitive and linguistic deficits have been found in up to 50% of the patients with BECTs (1, 2). Also, a deteriorate evolution of clinical manifestation and EEG has frequently been found in patients with atypical BECTs (3, 4).

The atypical evolution of BECTs may lead to atypical benign childhood partial epilepsy (ABPE), status epilepticus of BECTS (SEBECTS), Landau-Kleffner syndrome (LKS), and epileptic encephalopathy with continuous spike-and-wave during sleep (CSWS). These diseases are now considered to be different entities but part of a wide single spectrum of disorders named epileptic-aphasia spectrum (EAS), with BECTs situated at the mildest end having a relatively good prognosis while CSWS located at the most severe end has a mostly poor prognosis. EAS, a spectrum of epileptic, cognitive, and language disorders, is associated with (and presumably influenced by) the presence of electrical status epilepticus during slow-wave sleep (ESES) and imposes a catastrophic effect on the family and the growth of children (5–10).

Recently, some studies showed that children with EAS share some common epilepsy genes, including *GRIN2A* (glutamate receptor, ionotropic, *N*-methyl d-aspartate 2A), which is the most advocated one (7–15). Genetic identification of EAS offers the potential for understanding pathogenic mechanisms and targeted treatment options. For now, Gene Ontology (GO) analysis and protein-protein interaction (PPI) network related to EAS genes have never been studied and the study of genotype and phenotype relationship of EAS patients carrying pathogenic *GRIN2A* variants are currently a hot research area which has important value for early diagnosis and prognosis.

Furthermore, since ESES has been taken as the main object to explore the influence it has on cognition and prognosis in many previous studies, the potential pathogenic genetic factors attributing to cognitive function, treatment, and long-term prognosis have been studied poorly.

Therefore, in our research, we first summarized the electroclinical characteristics of children diagnosed with ABPE, LKS, or CSWS in our cohort. Secondly, we applied whole-exome sequencing (WES) test to six EAS children and epileptic panel to two EAS individuals whose parents were willing to cooperate and analyzed their genotype-phenotype relationships. Next, we summarized the manifestation features, treatments, and variant distribution of all the published literature reporting patients with *GRIN2A* missense mutations and truncation variants which led to NMDAR loss-of-function (LOF) change. In the end, the GO and PPI network of genes linked to EAS were further explored.

## Study Design

### Case Source

Patients diagnosed with ABPE, LKS, and CSWS were recruited from the neurological outpatient and inpatient units of the Chongqing Medical University's Affiliated Children's Hospital during January 2013 to June 2019. We retrospectively reviewed the clinical records and the EEG recording (including at least one entire sleep cycle) of all subjects.

### Inclusion and Exclusion Criteria

All subjects who possessed complete longitudinal clinical follow-up record, brain MRI results, EEG record, and at least one neuropsychological evaluation were included. Children with abnormal birth history and/or abnormal development history and/or significant abnormalities in brain MRI and/or abnormal hematuria metabolism screen results were excluded.

This study was approved by the Ethics Committee of the Chongqing Medical University's Affiliated Children's Hospital in China. Written informed consent was obtained from all the participants or their parents.

### Clinical Data Collection

Clinical information including gender, perinatal and family history, physical examination, age at the onset of seizure or ESES, seizure types, psychomotor evaluation before and/or after ESES, treatment, prognosis, metabolism screening, and neuroimaging results were collected through a review of clinical records combined with follow up telephone calls. Neuropsychological assessment: Wechsler Intelligence Scale for Children (WISC) and/or Test of Everyday Attention for Children (TEA-Ch) and/or Peabody Picture Vocabulary Test (PPVT) and/or Autism Behavior Check list (ABC) + Modified Checklist for Autism in Toddlers (M-CHAT) Scale were repeatedly performed during the admission and/or follow-up period.

### Definition (16)

#### ABPE

(1) The early course of ABPE is similar to BECTs, but patients exhibit more severe seizures, including epileptic negative myoclonus, atonic, atypical absence, myoclonic seizures, opercular syndrome, and frequent nocturnal focal seizures; (2) patients also suffer from mild neurocognitive and linguistic deficits; and (3) their EEG shows centro-temporal spikes (CTSs) which may develop into the ESES pattern. Classification: *type I*—Children with epileptic negative myoclonus and/or atypical absence and/or myoclonic seizures and/or frequent nocturnal focal seizures, manifesting as torpor, fine/gross motor clumsiness, drooping of the upper limbs head nodding, unexpected sudden falling, and mild cognitive impairment (17). *Type II*—Children with speech disorder/oropharynx manifesting as dysarthria, even complete aphasia, salivating, swallowing and tongue movement disorders when eating, and even opercular syndrome, such as dysphagia and bucking, without deficit in understanding speech and instruction (17). The intelligence of those children is usually normal.

#### LKS

(1) There is an abrupt or gradual onset of acquired language regression due to verbal auditory agnosia, with or without focal seizures, secondarily generalized tonic-clonic seizures, absences, or atonic seizures and (2) the EEG of LKS exhibits mainly bilateral synchronous or asynchronous temporal spikes which may develop into ESES.

#### CSWS

(1) Multiple types of seizures; (2) with moderate to severe global or selective regression affecting behavior, language, and intelligence; and (3) the EEG mostly shows ESES with a Spike-Wave Index (SWI) ≥50% during non-rapid eye movement (NREM) sleep.

#### ESES

EEG shows diffuse or bilaterally synchronous or non-synchronous spikes and waves at 1–3.5 Hz during NREM sleep, and the range of SWI is over 50% (The number of seconds containing epileptiform discharges was divided by the total number of seconds in the epoch, which was 600 s). EEG was recorded by scalp electrodes placed over the scalp according to the 10–20 International system. Children were divided into three groups, based on the SWI: group A (SWI ≥85%), group B (SWI 50–84%), and group C (SWI <50%).

#### Developmental Delay/Intellectual Disability (DD/ID)

DD/ID was evaluated according to the Diagnostic and Statistical Manual of Mental Disorders (DSM-5). DD/ID of patients were classified as follows: normal DD/ID = 0 points, borderline DD/ID = 1 point, mild DD/ID = 2 points, moderate DD/ID = 3 points, and severe DD/ID = 4 points. The terms DD and ID are used interchangeably here.

#### The Effects of AEDs

➀ seizure control: the seizure disappeared completely; ➁ seizure reduction: the frequency of seizures decreased ≥50%; and ➂ no effect: the frequency of seizures decreased ≤50%.

#### The Effects of Methylpyridines Pulse Therapy

➀ sensitive: SWI complete disappearance and response (SWI decreased to <50 or >20% reduction but still >50% or epileptic seizures completely disappeared); ➁ resistant: SWI did not change significantly (decreased <20%) or the reduction of seizures was <50%; ➂ dependent: SWI increased again or seizures reappeared/increased during or after the oral steroid withdrawal.

### Sequencing

A total of 535 genes known to be related to epileptic encephalopathy were included in our gene panel; the genes are listed in Supplementary Material. Two EAS individuals were conducted with epilepsy panel sequencing, while the rest six EAS patients with the WES. The processes for analyzing the sequencing data were as follows:

#### DNA Extraction

Genomic DNA was obtained from the peripheral blood of eight patients using standard methods and fragmented using Covaris sonicator (Covaris S2, USA).

#### Library Construction

Fragments were end repaired and adaptors were ligated using a standard Illumina paired-end (PE) adapter. Then, the ligated products were amplified through polymerase chain reaction (PCR). Subsequently, quality testing of the DNA library was performed through Nanodrop2000 and agarose gel electrophoresis, and the unqualified products were excluded.

#### Target Region Capture

Sequence capture, enrichment, and elution were performed following the manufacturer's instructions.

#### Sequencing

The final captured DNA libraries were analyzed by an Illumina HiSeq 2000 Sequencer following the manufacturer's sequencing protocols. The mean coverage and depth of all genes were 98% and 10 × average, respectively, which fulfilled the quality test.

#### Functional Annotation of Genetic Variants

The variants were annotated using public databases (Annotation Dependent Depletion (CADD) scores, HapMap database, dbSNP 144, ESP6500, and EXAC, 1,000 genome variants database) against the GRCh37 human reference genome and classified as missense, nonsense, splice-site, insertion, deletion, synonymous, or non-coding mutations.

All the novel variants were considered to be pathogenic if they met either: (1) stop/frameshift variants; (2) missense mutations situated in the amino acid conservative region across species; (3) splice-site variations fulfilling the GT-AT rules; or (4) predicted to be possibly damaging or disease causing by more than two bioinformatic programs, as follows: Sorting Intolerant from Tolerant (SIFT), PolyPhen-2, Mutation Taster, or Protein Variation Effect Analyzer (PROVEAN); {http://provean.jcvi.org/index.php}.

#### Sanger Sequencing

Sanger sequencing was performed to determine the variant validation and segregation analyses following standard protocols.

### Statistical Analysis

Continuous variables are reported as mean ± standard deviation. The Kruskal-Wallis test was used to analyze the correlation between the EEG-SWI and IQ level. Categorical variables are presented as frequencies and percentages. All analyses were conducted in SPSS 19.0 (SPSS Inc., Chicago, IL). The *t*-test and two-tailed *p*-values were used for all the analyses in this study, with *p* < 0.05 considered statistically significant.

## Results

From January 2013 to June 2019, 120 children were diagnosed with EAS in the Neurology Department of Chongqing Medical University's Affiliated Children's Hospital. Only 18 of them met the inclusion and exclusion criteria after our strict screening, and 33.3% (6/18) had LKS, 27.8% (5/18) had CSWS, and 38.9% (7/18) had ABPE. According to the ABPE classification method mentioned above, seven children with ABPE were divided into three type-I ABPE, three type-II ABPE, and one mixed-type ABPE. Gender ratio: (male:female = 5:4).

### Clinical Findings

#### Epilepsy

Sixteen out of eighteen children had seizure attacks, and their mean age at seizure onset was 55.4 ± 27.0 months (ranging from 27 to 107 months). Among that, 13 children's EEG showed ESES signal [ABPE (5), LKS (3), CSWS (5)]. The average time from seizure onset to ESES signal onset was 22.7 ± 15.5 months (ranging from 1 to 53 months). Seizure types are shown in Table 1. Eight patients had new epileptic attack forms within 3 months before or after the onset of ESES, in which five children had new types of seizure before ESES; three had it concurrently with ESES. The average time from seizure onset to appearance of new forms of attacks was 24.3 ± 15.2 months.

**Table 1:** Electroclinical features and diagnosis of 18 patients with EAS.

**No./Gender**	Family history^*^	**Age at SZ onset (months)**	**Age at last follow-up (months)**	**SZ types at onset (18)**	**New SZ types (months from SZ onset)**	**Behavioral disorder**	**ADHD**	**Cognitive impairment (Intel. Level/Lan. deficits/Learn. diff)**	**Motor dysfunction**	**EEG**		**Diagnosis**
										**Side and pattern**	**Max. SWI (months from SZ onset)**	
1F	No	42	144	GTCS	AA/AS (48)	Dull/irritability/attention deficit	NG	3/language delayed, slurred speech/learning difficulties, sensory integration disorder	Drooling, fine motor clumsiness, drooping of the upper limbs	Diffuse	≥85% (53)	CSWS
2M	Yes	77	156	GTCS	Absences/AS/eyes blink attack (40)	Irritability	NG	3/dysarthria/learning difficulties	Hypotonic, drooling, dysphagia	Multifocal	≥85% (41)	CSWS
3M	No	51	156	GTCS	AS (20)	NG	NG	3/auditory agnosia, expressive aphasia/learning difficulties	NG	Focal (L rolandic)	≥85% (24)	LKS + ESES
4M	Yes	107	151	GTCS	None	NG	NG	0/N/learning difficulties	NG	Diffuse	≥85% (1)	ABPE (I)
5F	No	80	122	FS/FBTCS/GTS/eyes blink attack	None	Attention deficit/slow reactiveness	NG	3/N/learning difficulties	Drooling, drooping of the upper limbs	Hemispheric (L)	≥85% (22)	CSWS
6F	No	67	117	GTCS	Absences (29)	Dull/irritability/attention deficit	NG	3/N/learning difficulties	Nocturnal urinary incontinence	Diffuse	≥85% (29)	CSWS
7F	No	72	108	GTCS/SE	AA/AS (24)	Attention deficit	NG	2/language regression/learning difficulties	Drooling	Focal (L rolandic)	50–84% (24)	ABPE (mix)
8F	No	35	72	FMS/FSIA	AA (23)	Slow reactiveness	NG	2/N/NG	NG	Diffuse	50–84% (23)	ABPE (I)
9M	No	28	67	FBTCS	None	Social interaction difficulty	NG	0/auditory agnosia, language regression/NG	Hypotonic, unwilling to walk	Focal (L rolandic)	<50%	LKS
10M	No	–	80	None	None	Slow reactiveness	NG	2/language regression, expressive aphasia/learning difficulties	Night terrors, poor sleep	Diffuse	≥85%	ABPE (II)
11M	Yes	27	97	FBTCS	None	Attention deficit	+	1/dysarthria/spatial-visual disorder	Tongue dyspraxia, drooling	Diffuse	50–84% (33)	ADHD+ABPE (II)
12F	No	30	110	GTCS	SE/FSIA (1)	NG	NG	3/language delayed, slurred speech/learning difficulties	NG	Diffuse	≥85% (3)	CSWS
13M	No	29	60	GTS/PMS/absences/AS	None	Social interaction difficulty, irritability with pinching people more often	NG	3/auditory agnosia, expressive aphasia/learning difficulties	Fine/gross motor clumsiness, negative myoclonus with sudden fall	Multifocal	<50%	LKS
14F	No	51	138	FSIA	None	Slow reactiveness	NG	2/auditory agnosia, expressive aphasia/learning difficulties	NG	Multifocal	50–84% (6)	LKS + ESES
15F	No	32	85	FS/GTS	SE/PMS/NMS/AS (9)	Slow reactiveness	NG	1/auditory agnosia, language regression/learning difficulties	Drooping of the upper limbs	Hemispheric (R)	≥85% (13)	LKS + ESES
16M	Yes	100	168	GTCS	None	Attention deficit/hyperactive	+	1/N/learning difficulties	Night terrors, poor sleep	Diffuse	<50%	ABPE (I) + ADHD
17M	No	–	138	None	None	NG	NG	0/speech dyspraxia, slurred speech/*N*	Tongue dyspraxia	Focal (R rolandic)	<50%	ABPE (II)
18M	No	32	72	FBTCS/PMS/AS	None	Social interaction difficulty	NG	3 auditory agnosia, language regression/learning difficulties	NG	Multifocal	<50%	LKS

F, female; M, male; N, normal; SZ, seizure; FMS, focal motor seizures; FS, focal seizures; FSIA, focal seizures with impaired awareness; FBTCS, focal to bilateral tonic-clonic seizures; SE, status epilepticus; GTCS, generalized tonic-clonic seizures; GTS, generalized tonic seizures; AA, atypical absences; PMS, positive myoclonic seizures; NMS, negative myoclonic seizures; AS, atonic seizures; DD/ID, developmental delay/intellectual disability (normal IQ or no DD/ID = 0 points, borderline DD/ID = 1 point), mild DD/ID = 2 points, moderate DD/ID = 3 points, severe DD/ID = 4 points); SWI, spike-wave index; R, right; L, left; LKS, Landau-Kleffner syndrome; CSWS, epileptic encephalopathy with continuous spike-and-wave during sleep; ABPE, atypical benign partial epilepsy; ADHD, attention-deficit hyperactivity disorder; ESES, electrical status epilepticus during slow-wave sleep; CZP, clonazepam; VPA, sodium valproate; LEV, levetiracetam; LTG, lamotrigine; OXC, oxcarbazepine; NZP, nitrazepam; Intel. Level, intellectual level; Lan. deficits, language deficits; Learn. diff, learning difficulties.

* *Epilepsy/febrile seizures*.

Two children had no seizure (patients No. 10 and No. 17). According to their clinical manifestation, with aphasia and oropharyngeal dyskinesia with normal auditory tests, they were diagnosed as ABPE type II. The EEG signal of patient No. 10 showed a diffuse spike-slow wave in the bilateral center and posterior temporal area (SWI ≥ 85%), while the EEG of patient No. 17 showed a focal spike-slow wave in the rolandic area on the right hemisphere (SWI <50%).

#### Cognitive/Behavioral/Motor Symptoms

The cognitive, behavioral, and motor function impairment of 18 children are shown in Table 2. Among the 13 patients with ESES, 10 (76.9%) presented one or more behavioral abnormalities, such as dullness, irritability, aggressive behavior, attention deficit, and slow reactiveness, and one was diagnosed as having attention-deficit and hyperactivity disorder (ADHD). All patients (100%) with ESES showed cognitive impairment, which presented as intellectual impairment [1/13 (7.7%) normal, 2/13 (15.3%) borderline, 4/13 (30.8%) mild, 6/13 (46.2%) moderate to severe], learning difficulties, visual motor/visual spatial impairment, sensory integration disorder, and language impairment. Eight of 13 patients (61.5%) had motor dysfunction, such as decreased motor coordination, fine/gross motor clumsiness, orofacial dyspraxia with drooling, negative myoclonus with sudden falls, and negative myoclonus with drooping of the upper limbs.

**Table 2:** The cognitive, behavioral, and motor function impairment of 18 children.

**No**.	**SZ types (18)**	**AEDs before steroid**	**Effect**	**Steroid**	**Effect**	**AEDs before steroid**	**Effect**	**Prognosis**	
1F	GTCs/AA/AS	VPA/LEV/NZP/TPM	No effect	Resistant	No effect	Clobazam/KD	Seizure reduced	Behavior/cognition/motor have been improved but not returned to normal	Seizure reduction, EEG not normalized
2M	GTCs/absences/AS	VPA/LTG/CZP	No effect	Dependent	Seizure control/SWI decreased; seizure reduction/SWI decreased	NZP/VPA/LEV	Seizure reduced	Learning difficulties, bad memory, but language back to normal	Seizure control, EEG not normalized
3M	GTCs/AS	VPA/OXC/LEV/NZP	No effect	Resistant	No effect	KD	Seizure controlled	All back to normal	Seizure control; EEG normalized
4M	GTCs	LEV	Seizure reduced	dependent	Seizure reduced, SWI decreased (2 times)	LEV	Seizure controlled	All back to normal	Seizure controlled, EEG normalized
5F	FBTCS/GTS/eyes blink attack/FS	VPA/NZP/LEV	Seizure reduced	–	–	–	–	Learning difficulties, drooping of the upper limbs, slow reactiveness	Seizure not controlled, EEG not normalized
6F	GTCs/absences	VPA/LEV/CZP	Seizure reduced	Sensitive	Seizure controlled, SWI decreased	VPA/LEV	Seizure controlled	Learning difficulties, slow reactiveness, bad memory	Seizure controlled, EEG not normalized
7F	GTCs/SE/AA/AS	LEV/VPA/NZP	Seizure reduced	Sensitive	Seizure controlled, SWI decreased	OXC	Seizure controlled	All back to normal	Seizure controlled, was not re-examined timely
8F	FSIA/FMS	VPA/CZP/LEV	Seizure reduced	–	–	–	–	All back to normal	Seizure not controlled, EEG not normalized
9M	FBTCS	LEV/OXC	Seizure controlled	–	–	–	–	Poor verbal expression, incoherent, and slurred speech	Seizure controlled, was not reexamined timely
10M	None	LEV	–	Sensitive	SWI decreased	–	–	All back to normal	EEG normalized
11M	FBTCS	VPA	Seizure controlled	–		–	–	Cognition improved, but tongue dyspraxia and salivation remained	Seizure controlled, EEG not normalized
12F	GTCs/SE/FSIA	LEV/OXC/CZP	Seizure reduced	Resistant	SWI decreased	VPA/NZP/DZP	Seizure reduced	NG	Seizure not controlled, EEG not normalized
13M	GTS/PMS/absences/AS	VPA/NZP/CZP	Seizure reduced	–	–	KD	Seizure reduced	Cognition and language improved, but not back to normal, poor verbal expression, and slurred speech	Seizure not controlled, EEG not normalized
14F	FSIA	VPA	No effect	Sensitive	Seizure controlled, SWI decreased	LEV	Seizure controlled	All back to normal	Seizure controlled, EEG normalized
15F	GTS/FS/SE/PMS/NMS/AS	VPA/NZP/LEV	No effect	Dependent	Seizure reduced, SWI decreased (3 times)	VPA/LEV/NZP	Seizure reduced	Cognition and language improved, but not back to normal	Seizure controlled, EEG normalized
16M	GTCS	VPA	Seizure controlled	–	–	–	–	NG	Seizure controlled, EEG normalized
17M	None	–	–	–	–	–	–	NG	Was not reexamined timely
18M	FBTCS/PMS/AS	OXC/CZP/VPA	Seizure reduced	Sensitive	Seizure controlled	–	–	All back to normal	Seizure controlled, EEG normalized

*NG, not given; N, normal; F, female; M, male; SWI, spike-wave index; FMS, focal motor seizures; FSIA, focal seizures with impaired awareness; FBTCS, focal to bilateral tonic-clonic seizure; GTCS, generalized tonic-clonic seizures; GTS, generalized tonic seizures; AA, atypical absences; PMS, positive myoclonic seizures; AS, atonic seizures; CZP, clonazepam; VPA, sodium valproate; LEV, levetiracetam; LTG, lamotrigine; OXC, oxcarbazepine; NZP, nitrazepam; DZP, diazepam; KD, ketogenic diet*.

Among the five patients without ESES, four (80%) manifested behavioral abnormalities, such as attention deficit, hyperactivity, irritability, and impaired social interactions, and one was also diagnosed with ADHD. All patients without ESES (100%) had cognitive impairment, which presented the same as what was mentioned above in the group of patients with ESES, but a different degree (intellectual impairment: 2/5 (40%) normal, 1/5 (20%) borderline, 2 (40%) moderate to severe). Four of five patients (80%) had motor dysfunctions, such as fine/gross motor clumsiness, dysarthria, tongue dyspraxia, hypotonia, negative myoclonus with sudden fall, and night terrors.

A total of 13/18 (72.2%) children had language impairment. Global intelligence quotient (IQ) ranged from 41 to 96 in the entire sample, with an average score of 66.7 ± 17.0. Neuropsychiatric comorbidity was found in two individuals with ADHD (10%).

Notably, in our study, ID of children diagnosed with LKS ranged from normal to moderate-severe (Table 3), and two who had moderate-severe ID had EEG-SWI <50% while one with borderline IQ had EEG-SWI ≥85%.

**Table 3:** SWI and IQ or DD/ID characteristics of 18 patients.

**EEG**\**ID**	**Normal IQ (0)**	**Borderline IQ (1)**	**Mild DD/ID (2)**	**Moderate to severe DD/ID (3)**	**Sum**
SWI <50%	2	1	0	2	5
SWI 50–84%	0	1	3	0	4
SWI ≥85%	1	1	1	6	9
Sum	3	3	4	8	18

*SWI, spike-wave index; IQ, intelligence quotient; DD/ID, developmental delay/intellectual disability*.

### Accessory Examination

#### Brain MRI

All of the 18 patients went through MRI. Four patients had subtle demyelination anomalies (No. 3, No. 4, No. 11, and No. 18), and the brain MRIs of the remaining individuals were normal. However, after the reassessment of two neurologists, the mild abnormality in the four patients' MRI was found to be of no clinical significance.

#### Twenty-Four-Hours EEG

In our study, 50% (9/18) of the individuals had an EEG-SWI ≥85%, 22.2% (4/18) had an EEG-SWI between 50 and 84%, and 27.8% (5/18) had an EEG-SWI <50%. The baseline severity of the SWI in groups A, B, and C was not significantly correlated with their DD/ID severity (rho = 0.364, *p* = 0.138, *n* = 18) (Table 4). Notably, the deterioration of clinical manifestation (including the onset of new seizures or appearance of behavioral/cognitive/motor impairment) in children with ESES all appeared before the onset or at the onset of their ESES.

**Table 4:** Intelligence distribution of children diagnosed as ABPE/LKS/CSWS.

**Diagnosis**	**Cases**	**Intelligence level**			
		**Normal (0)**	**Borderline (1)**	**Mild (2)**	**Moderate–severe (3)**
ABPE	7	2	2	3	0
LKS	6	1	1	1	3
CSWS	5	0	0	0	5

### Pathogenic Variants

2 of the 8 patients (25.0%) who were sequenced were found to carry *de novo* pathogenic *GRIN2A* mutations (BioSample accessions: SRR12681660, SRR12681661). These two *GRIN2A* mutations were detected in two male patients with ABPE (II) patient No. 10 (Proband 1): cDNA c.2094T>G (p.Y698^*^) and patient No. 11 (Proband 2): cDNA c.2041C>T (p.Y681^*^) and were found to be pathogenic according to the guidelines of the American College of Medical Genetics and Genomics (ACMG) (the evidence levels of the two variants were both PVS + PS). Patient No. 9 was found to carry an inherited *GRIN2A* mutation: cDNA c.4126C>T (p.R1376C) which was ruled out later because of no segregation found in the proband's family (even Mutation Taster and PolyPhen-2 outcomes showed “disease causing”). The mutation of gene *SLC2A1*: cDNA c.668G>A (p.R223Q) was identified in patient No. 1 who was diagnosed with CSWS and had cerebrospinal fluid (CSF)/periphery blood glucose ratio of 0.56 (borderline CSF glucose for children, 2.7 mmol/l). However, this mutation was ruled out later as well, because the functional experiment of R233Q had been performed in 2012 with an outcome as “no functional change.”

Proband 1 (Tyr698^*^) is a male aged 6.8 years old with a negative family history and a normal perinatal period. He could sing children's songs and had normal neuromotor development before becoming ill. His language regression initially started at 3 years and 11 months as he gradually lost the ability to speak or only unconsciously mumbled “BABA” in 1 month. However, he did not have any difficulty in following auditory instructions. His Wechsler Intelligence Scale score was 70 points. He did not have any seizures, but with ESES affecting centrotemporal area on the left side and parietal, occipital, and temporal lobes on the right side. Brain MRI was normal. Levetiracetam combined with a high dose of intravenous methylprednisolone (two episodes) was given when he was 4 years old, and subsequently, the linguistic impairment and intellectual deficit gradually improved.

Proband 2 (Arg681^*^) is a male aged 8.1 years old with normal perinatal period and a positive family history, as his father had febrile seizure once during childhood. The proband suffered recurrent partial seizures (including focal to bilateral tonic-clonic seizures) which occasionally happened after febris or afebris, six times in total and lasting 10–30 min per time since he was 2 years and 3 months old. His score on the Wechsler Intelligence Scale was 85 points, whereas neuropsychological evaluation presented spatial-visual disorder, expressive language impairment, and ADHD. The EEG showed a spike-slow wave on both sides of the front and the middle temporal lobe areas during the wake-up and sleeping period, especially during the sleeping period. MRI result presented abnormal signal on the right-side temporal lobe at the age of 2.5 but was normal after being rescanned at 4 and 7 years old, respectively. He was treated with valproic acid around the age of 3 years old, and after that, his seizures relieved in 60 months while language impairment remained.

**Figure 1:**
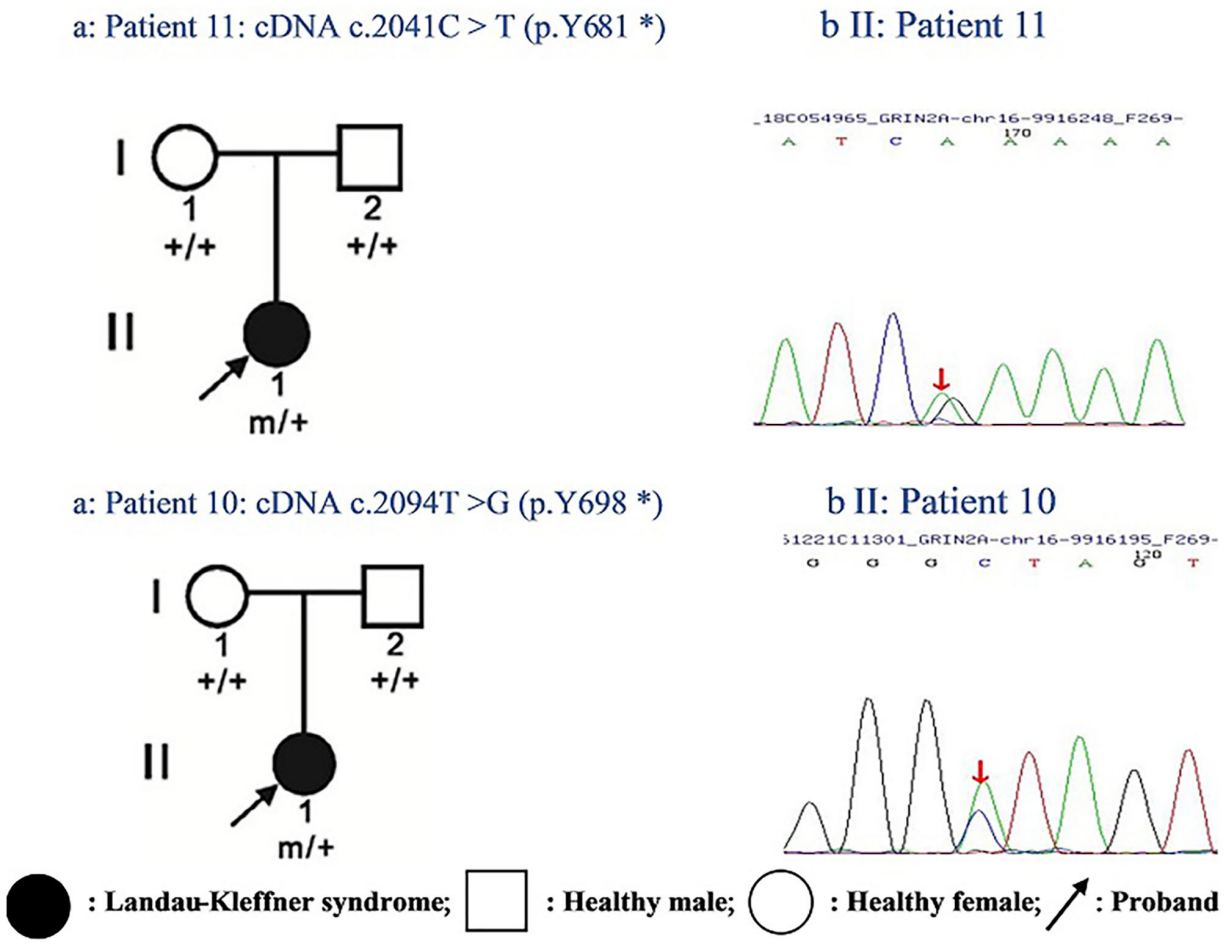
Pedigrees of two cases with epilepsy-aphasia disorders and identified *GRIN2A* mutations: **(a)** Filled-in circles indicate individuals with Landau-Kleffner syndrome; empty circles/squares indicate unaffected women/men. Arrows indicate the probands. Individuals with the *GRIN2A* mutation are indicated by m/+, and mutation-negative individuals are indicated by +/+. **(b)** Chromatograms of *GRIN2A* mutation detected in two probands. Arrows show the position of the mutation.

**Figure 2:**
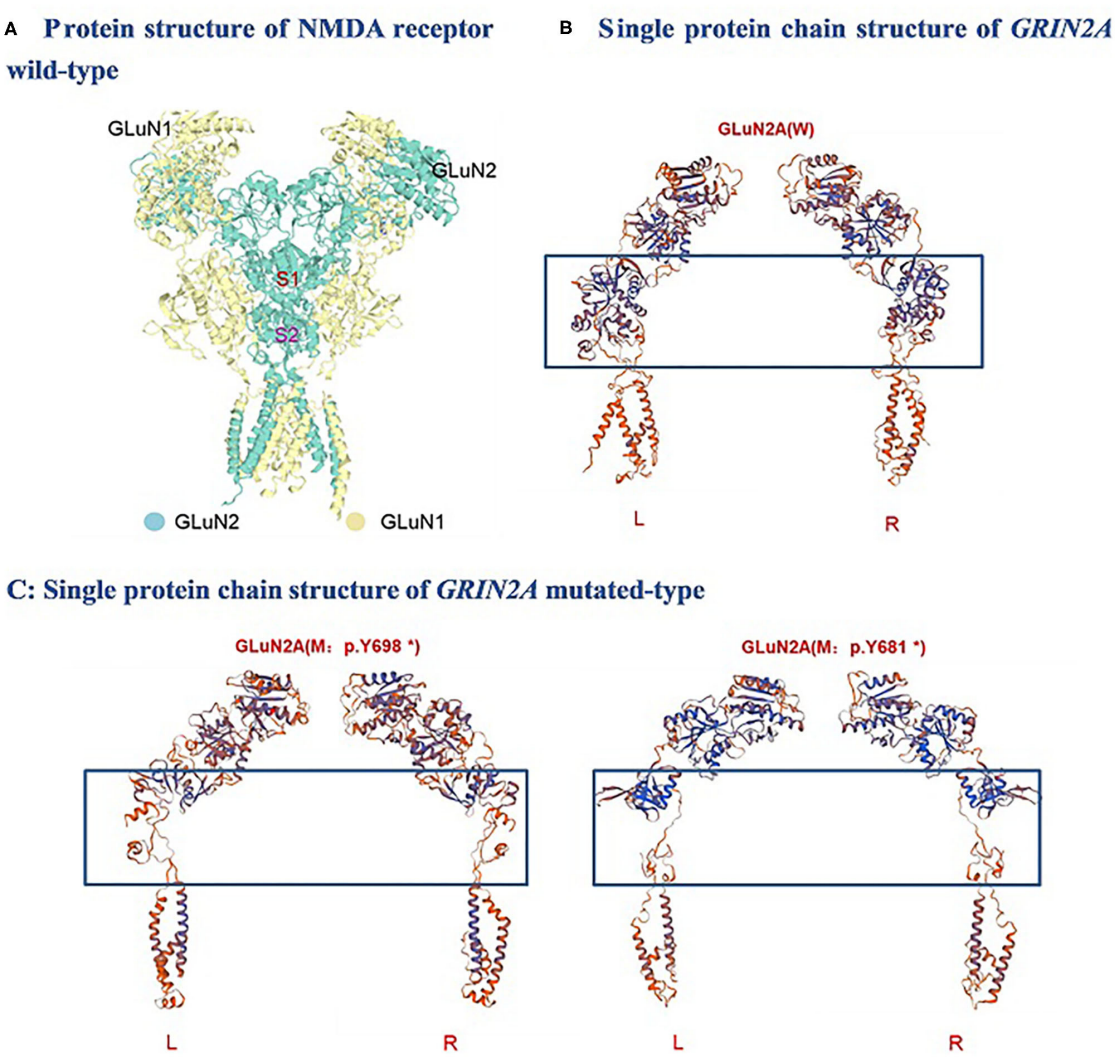
Molecular analysis of *GRIN2A* variants: 3-D protein chain structural changes in *GRIN2A* mutated-type compared with the wild-type were predicted with SWISS-MODEL. S1, S1 ligand-binding domains; S2, S2 ligand-binding domains; NMDA, N-methyl-D-aspartate; W, Wild-type; M, mutated-type; L, Left; R, Right.

One of the identified pathogenic *GRIN2A* mutations-*de novo* c.2094T>G (p.Y698^*^) has not been reported before, whereas the *GRIN2A* mutation-*de novo* c.2041C>T (p.Y681^*^) has been reported in a family with LKS in 2013 (inherited). Both of the *GRIN2A* variants led to the end of the protein transcription/translation and caused NMDAR loss-of-function change (Table 5; Figure 1). Three-dimensional protein chain structures of these two variants were constructed with SWISS-MODEL, which showed both of the variants affecting the S2-LBD area (Figure 2).

**Table 5:** Genetic and protein change data of patient 10 and patient 11.

**Proband**	**Chr**	**Positon (bp)**	**cDNA change**	**Protein change**	**Homo/Hete**	**Inher**	***GRIN2A* domain**	**Phenotype**	**References**
Patient 10 (Proband 1)	16	9,916,195	c.2094T>G	p.R698^*^	Hete	*De novo* exon 11	S2LBD	ABPE-II	None
Patient 11 (Proband 2)	16	9,916,248	c.2041C>T	p.Y681^*^	Hete	*De novo* exon 11	S2LBD	ABPE (II) + ADHD	(7)

*Chr, chromosome; CDNA, complementary DNA; Homo, homozygous; Hete, heterozygous; ABPE, atypical benign childhood partial epilepsy; ADHD, attention-deficit hyperactivity disorder*.

### Treatment

#### AEDs

In this cohort, 16 patients had seizures and 17 patients were treated with 1–5 (average 2.8) different types of AEDs. Among them, only 2 (12.5%, 2/16) patients had their seizures controlled with monotherapy, and only one had EEG normalization. Three (18.8%, 3/16) patients received therapy with multiple AEDs, and only one of which reached controlled seizures while none of which had EEG normalization. Eleven (68.7%, 11/16) patients were treated with multiple AEDs combined with steroids, and six had their seizures controlled while four had EEG normalization. In addition, three patients who were resistant to AEDs or steroid therapy were treated with a ketogenic diet (KD) subsequently, and one of them had their seizures controlled, EEG normalized, and neuropsychological function recovery (2 years after the ketogenic diet was introduced) eventually. Moreover, the other two children treated with KD also had their seizures and EEG significantly improved 4–8 months later.

In this study, nine different kinds of AEDs were added, including valproate, levetiracetam, nitrazepam, topiramate, lamotrigine, clonazepam, oxcarbazepine, clobazam, and diazepam. The anticonvulsants initially used in this cohort were mostly valproic acid (64.7% of patients), levetiracetam (29.4%), and oxcarbazepine (5.9%). The most effective (seizure control + seizure reduction) AEDs were valproate (4/17, 23.5%), valproate + nitrazepam/clonazepam (5/17, 29.4%), and levetiracetam (2/17, 11.8%). It should be noted that there were two children who suffered from absence after the addition of levetiracetam.

#### Steroids

Of the 11 patients who received steroid treatment because their SWI continued to increase despite taking AEDs, 45.4% (5/11) were sensitive to steroids, 27.3% (3/11) were resistant to steroids, and 27.3% (3/11) were steroid-dependent.

#### Prognosis

Until August 2019, the average time of follow-up was 61.7 ± 26.4 months (31–114 months). For 16 patients who had seizures, 11 were seizure free, and seven had EEG normalization. The mean time from the onset of attack to being seizure free was 27.5 ± 23.3 months (*n* = 11), and the mean time for the EEG to return to normal was 37 ± 25.4 months (*n* = 7). The cognitive/behavioral/motor functions were all back to normal in seven patients. Among the patients who were seizure free at the last follow-up, five still suffered from different kinds of deficits. However, in five cases, their cognitive/behavioral/motor functions almost returned to a normal level after EEG normalization (Table 2).

## Literature Review of Pathogenic *GRIN2A-*Lof Variants

We reviewed all the published literature reporting patients with pathogenic *GRIN2A* variants, and missense mutations which were proved to result in NMDAR-LOF change were selected from those literatures along with *GRIN2A* truncation variants (Table 6). There were 18 *GRIN2A* missense mutations and 11 *GRIN2A* truncating mutations in total which lead to NMDAR-LOF change. Of these mutations, 9 (31.0%) were situated in NTD, 6 (20.7%) in LBD (S1), 10 (34.5%) in LBD (S2), 2 (6.9%) in CTD, and only 2 (6.9%) in TMD and linker domain, respectively. Among those literatures we reviewed, the language situations of 22 cases were descripted in detail; 95.5% of them manifested language deficit, such as speech/language developmental delay, language regression, acquired aphasia, auditory agnosia, verbal dyspraxia, slurred speech, and dysarthria. Among the 14 cases which had clear intellectual evaluation in the literatures, 28.6% had normal IQ, 21.4% had mild ID/DD, 28.6% had moderate ID/DD, 14.3% had mild-moderate ID/DD, and only 7.1% had severe ID/DD. To the best of our knowledge, the treatment process of only two patients has been described in detail in all of the published literature in Table 6 (11, 22). One of the two patients diagnosed with LKS were treated with prednisone and immunoglobin after being unresponsive to multiple AEDs, and the cognition of this patient improved gradually 6 months after prednisone and immunoglobin treatment. Another patient diagnosed with atypical rolandic epilepsy was treated with four AEDs but responded poorly. Both of their EEGs showed ESES.

**Table 6:** Characteristics of patients with *GRIN2A*-LOF variants (missense + truncation).

***GRIN2A***	**Protein**	**Gene**	**Hete/Homo**	**Origin**	**Domain**	**Seizure onset**	**ID/DD onset**	**Others**	**Phenotype**	**EEG**	**Diagnosis**	**Treatment**	**References**
Missense	Pro79Arg	c.236C>G	Het	Inherited	NTD	6 years	Mild	Speech developmental delay	Focal seizures	ESES	CSWS + ADHD; BECTs (mother, uncle, grandmother)	NG	(7)
Missense	lle184Ser	c.551T>G	Het	Inherited	NTD	4 years	NG	Acquired aphasia, cognitive delayed	Absence, atonic	ESES	CSWS	NG	(9, 19)
Missense	Cys231Tyr	c.692G>A	Het	Inherited	NTD	3 years	Mild/no	Speech developmental delay, mild hypotonic	Focal seizures with secondary generalization	ESES	LKS + ID (proband); CTS (brother, sister)	NG	(7)
Missense	Cys436Arg	c.1306T>C	Het	*De novo*	LBD (S1)	4 years	NO	Speech developmental delay	Generalized seizures	Focal (CTS)	ABPE	NG	(20, 21)
Missense	Gly483Arg	c.1447G>A	Het	Inherited	LBD (S1)	4 years	NG	Language delayed, cognitive globally delayed	Focal seizures/no seizure	ESES/CTS	CSWS/ARE + dysphasia	NG	(9, 20)
Missense	Arg504Trp	c.1510C>T	Het	Inherited	LBD (S1)	4 years	NG	Language normal, cognitive delayed VIQ = 47, PIQ = 41	Complex partial seizures	ESES	CSWS+ADHD	NG	(9)
Missense	Arg518His	c.1553G>A	Het	Inherited	LBD (S1)	2 years/3 years	NG	Language delayed	Focal seizures/no seizures	ESES	CSWS/LKS/CSWS + verbal dyspraxia/atypical rolandic epilepsy + verbal dyspraxia/verbal dyspraxia “only”	NG	(9, 19)
Missense	Thr531Met	c.1592C>T	Het	Inherited	LBD (S1)	NG	Moderate/severe	Speech/language impairment	Focal seizures	CTS/ESES	CSWS/epilepsy-aphasia/learning difficulty + speech/language impairment	NG	(1)
Missense	Ala548Thr	c.1642G>A	Het	*De novo*	Linker	6 years	Moderate	Language regression, cognitive delayed, VIQ = 53, PIQ = 45	Focal seizures	ESES	LKS + ADHD	NG	(9)
Missense	Val685Gly	c.2054T>G	Het	NG	LBD (S2)	NG	NG	Global developmental delay	Severe intractable epilepsy	NG	NG	Pregnenolone sulfate enhanced the GluN2A-LOF activity.	(20)
Missense	lle694Thr	c.2081T>C	Het	*De novo*	LBD (S2)	2.5 years	NO	Language regression, VIQ = 89, PIQ = 84	Complex Focal seizures	ESES	LKS	NG	(7)
Missense	Met705Val	c.2113A>G	Het	Inherited	LBD (S2)	8 years	NO	Speech developmental delay	Focal seizures with secondary generalization	CTS	BECTs	NG	(7, 20)
Missense	Glu714Lys	c.2140G>A	Het	NG	LBD (S2)	NG	NG	NG	NG	NG	CSWS	NG	(20)
Missense	Ala716Thr	c.2146G	Het	*De novo*	LBD (S2)	2 years	NG	Speech regression, unable to understand first complex and later simple commands, diffuse hypotonia, motor coordination disorder	Focal seizures	ESES	LKS/ADHD + apraxia/ADHD + seizures	LEV + DZP language and EEG improved, but relapse while weaning off, prednisone + IVIG (speech gradually improved over 6 months)	(20, 22)
Missense	Ala716Thr	c.2146G	Het	Inherited	LBD (S2)	NG	NG	10 family members with inherited p.Asn327Ser *SRPX2* alteration the same time had verbal dyspraxia and cognitive impairment	NO	NG	ABPE	NG	(9)
Missense	Ala727Thr	c.2179G>A	Het	NG	LBD (S2)	NG	NG	NG	NG	NG	BECTs	NG	(20)
Missense	Asp731Asn	c.2191G>A	Het	*De novo*	LBD (S2)	4 years/5 years	1 year	Cognitive developmental delay, verbal dyspraxia, paroxysmal weakness of right lower limb, gait abnormality	Focal seizures (restricted to periods of sleep)/focal seizures, GTCS (resolved in late childhood)	ESES/CTS	Atypical rolandic epilepsy/LKS	4 AEDs LEV/CZP partially effective/NG	(11, 20, 23)
Missense	Asp731Asn	c.2191G>A	Het	Inherited	LBD (S2)	2 years	NG	Verbal dyspraxia, intellectual, motor, and cognitive regression, and language delay	GTCS	CTS	Atypical rolandic epilepsy + VD	NG	(9)
Missense	Val734Leu	c.2200G>C	Het	Inherited	LBD (S2)	NG	NG	NG	NG	NG	BECTS	NG	(20)
Missense	Lys772Glu	c.2314A>G	Het	NG	LBD (S2)	NG	NG	NG	NG	NG	ABPE + learning and reading problems	NG	(20)
Truncating mutation	Trp55^*^	c.165G>A	Het	Inherited	NTD	NG	NG	NG	NG	NG	NG	NG	(24)
Truncating mutation	Gln163^*^	c.487C>T	Het	Inherited	NTD	NG	NG	NG	NG	NG	NG	NG	(24)
Truncating mutation	Trp198^*^	c.594G>A	Het	NG	NTD	3.5 years	NG	Milestones delayed, mild hypotonia, cognitive delayed, language delayed, slurred speech	Atonic seizure	CTS	ABPE	NG	(24)
Truncating mutation	Gln 218^*^	c.652C>T	Het	Inherited	NTD	NG	Mild to moderate	Learning difficulties (2)/behavioral abnormality, muscular *hypotonia*	Seizures within 1 year, relieved in adolescence/complicated febrile seizures started at 11 months, focal seizures until age of 3 years/no seizure	ESES	NG	NG	(7)
Truncating mutation	Leu334^*^	c.1001T>A	Het	Inherited	NTD	4 years	NG	Mild fine motor clumsiness, cognitive lower normal range, language delayed	Rolandic seizure	ESES	Panayiotopoulos/CSWS/partial epilepsy	NG	
Truncating mutation	Lys346^*^	c.1036A>T	Het	*De novo*	NTD	NG	Mild	ID/DD, mild hypotonia	No seizure	Multifocal	NG	NG	(9)
Truncating mutation	Ser538^*^	c.1613C>G	Het	*De novo*	LBD (S1)	4 years	Moderate	ID/DD, dysarthria, mild ataxia	Focal seizures with secondary generalization	ESES	NG	NG	(15)
Truncating mutation	Trp606^*^	c.1818G>A	Het	*De novo*	TSM	3 years and 6 months	Moderate	ID/DD, speech developmental delay, ataxia	Focal seizures	Multifocal	NG	NG	(15)
Truncating mutation	Arg681^*^	c.2041C>T	Het	Inherited	LBD (S2)	3 years and 6 months	NG	Auditory agnosia, receptive + expressive language disorder, learning disability	No seizures	ESES	LKS	NG	(15)
Truncating mutation	Tyr943^*^	c.2829C>G	Het	Inherited	CTD	2 years	Mild-moderate	Fine/gross motor clumsiness, cognitive retardation, language delayed	Rolandic seizure	ESES	CSWS	NG	(15)
Truncating mutation	Tyr1387^*^	c.4161C>A	Het	Inherited	CTD	4 years	NG	Language delayed	GTCS Proband's mother her brother both had seizures in childhood that resolved before adulthood	ESES	CSWS + autistic features + ADHD	NG	(15)

*NG, not given; NTD, amino (N)-terminal domain; LBD, linger-binding domain; TMD, transmembrane domain; CTD, intracellular C-terminal domain; ABPE, atypical benign partial epilepsy; ARE, atypical rolandic epilepsy; BECTs, benign epilepsy with centrotemporal spikes; CSWS, epileptic encephalopathy with continuous spike-and-wave during sleep; CTS, centrotemporal spikes; ESES, electrical status epilepticus during slow-wave sleep; DD, developmental delay; LOF, loss of function; GTCS, generalized tonic-clonic seizures; LKS, Landau-Kleffner syndrome; VIQ, verbal intelligence quotient; PIQ, operative intelligence quotient; Het, heterozygote; Homo, homozygote*.

## Correlation Analysis of EAS-Related Genes

**Figure 3:**
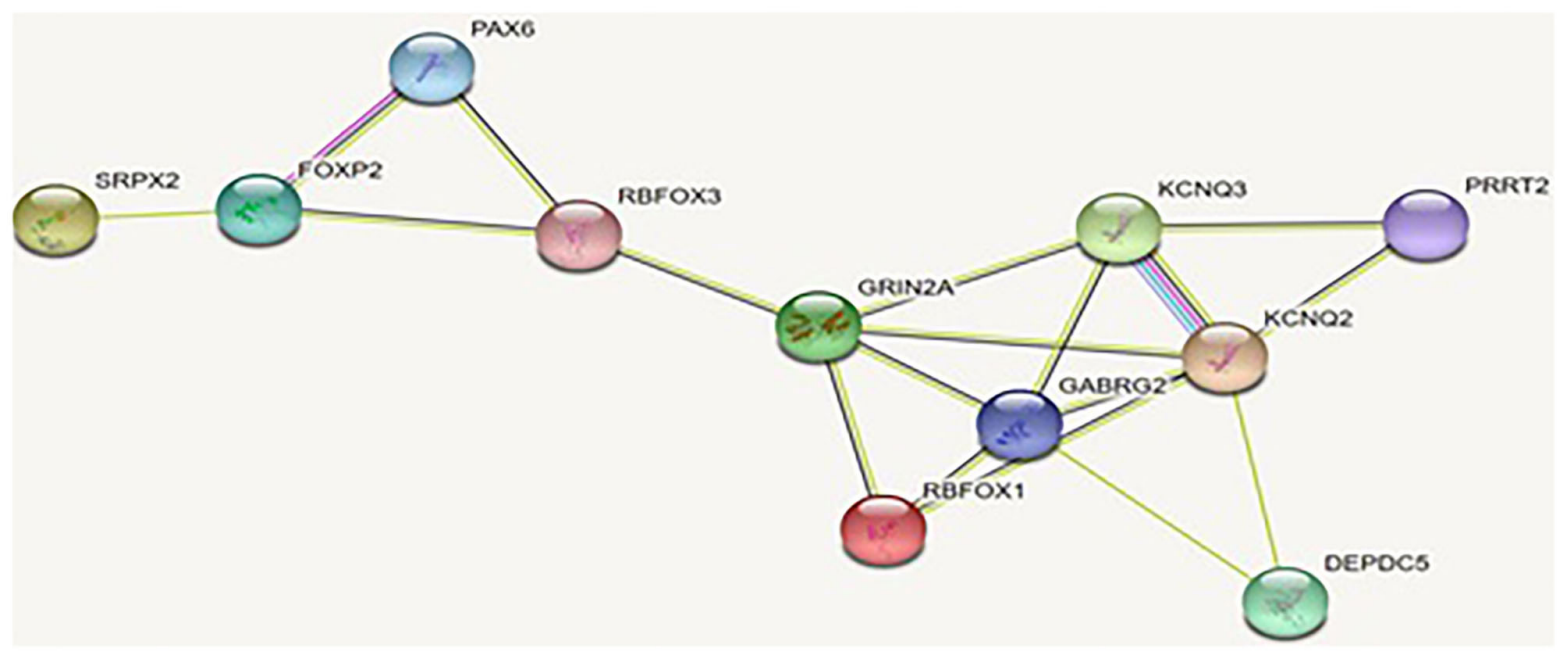
Interaction of the proteins encoded by the genes related to EAS: the interaction of these proteins is stronger than a group of similar size proteins randomly selected from the genome. Such enrichment suggests that these proteins are biologically related and that the *GRIN2A* gene appears to be the hub gene of PPI.

We conducted systematic literature review to identify all of the EAS-related genes in our previous work (25). In this study, we applied string software to study the PPI of those EAS-related genes, and the results are shown in Figure 3.

Further, we analyzed the gene ontology to understand the biological process, molecular function, and cellular components [false-discovery rate (FDR), <0.01] in EAS-related genes. The results are shown in Table 7.

**Table 7:** Gene ontology of genes related to EAS.

**Biological process**	**FDR**	**Molecular function**	**FDR**	**Cellular component**	**FDR**
Chemical synaptic transmission	0.0011	Voltage-gated cation channel activity	0.0032	Neuron part	0.0018
Vocalization	0.0060	Ion-gated channel activity	0.0032	Synaptic membrane	0.0018
–	–	Ion channel activity	0.0032	Axon initial segment	0.0018
–	–	–	–	Ion channel complex	0.0018

## Discussion

### Electroclinical Characteristics

Epilepsy is the most typical feature in our group, and the average age of onset was 4.5 ± 2.2 years, which is significantly earlier than that of a previous large-scale study of Chinese classic BECTS (6.85 ± 2.45 years, 1,817 children) (26).

In our group, the onset of new seizures or the appearance of behavioral/cognitive/motor impairment in children with ESES all appeared earlier or at the same time as the onset of their ESES. To the best of our knowledge, there are few research studies on the time relationship between ESES and clinical deterioration.

Therefore, this data might suggest that when children with suspected BECTs have an early onset, changed seizure semiology, and deterioration of behavior/cognition/motor function, neurologists should be alert on the appearance of ESES.

Now, there are inconsistent agreements on the relationship between the EEG SWI severity and the degree of cognitive impairment in children with EAS. Previous studies have reported that the severity of cognitive impairment in EAS patients seem to be related to the EEG SWI, but in recent years, some studies have disagreed with that. Wolff et al. reported that cognitive deficits were associated with spike location but not with the SWI in 20 children with IFE (27), whereas Nicolai et al. reported that the correlation between cognitive deficits and EEG location was not consistent (28). Overvliet et al. found that there was a relationship between the cognitive deficits and SWI in nocturne, which indicated that cognitive and behavioral functional deficits were concordant with the magnitude of the EEG discharge index (29). In our study, however, the baseline severity of the SWI was not significantly correlated with the DD/ID severity. The possible explanation might be as follows: first, the neuropsychological evaluation did not always parallel with the EEG evaluation due to the poor compliance of some patients. Furthermore, inflammation may be one of the mechanisms. In 2016, Munckhof et al. found that the levels of IL-1A, IL-6, and CXCL8/IL-8 of patients with ESES were significantly higher than those of the control group. After immunotherapy (steroid or immunoglobulin) was given, the level of IL-6 decreased significantly. The change of IL-6 was accompanied by the improvement of EEG SWI and neuropsychological evaluation (30). Third, sleep structure disruption may also contribute to the cognitive deficit. Sleep has been demonstrated to play an important role in neurophysiological and neurochemical processes underlying the memory and learning consolidation (31). Epilepsy and pathognomonic sleep-related EEG pattern as ESES in EAS may impair patients' cognition by affecting normal sleep-awareness cycles, resulting in decreased total sleep time, lower sleep efficiency, and higher arousal index. Moreover, sleep fragmentation disorders could further aggravate the seizure situation as a vicious circle (32). However, the sleep quality evaluation of patients in this study has not been performed due to limited recording instrument and poor compliance. Finally, genetic factors might contribute to the intellectual impairment in some EAS children.

In conclusion, ESES is limited in estimating the degree of cognitive deficits because the deterioration of clinical manifestations was ahead of or synchronized with the ESES occurrence in our study and the severity of the SWI did not parallel with the DD/ID severity either. Therefore, we hypothesize that the neuropsychological deterioration in children with EAS is not only completely affected by electric discharge but also may be affected by autoimmune response, sleep disorder, and genetic etiology. However, the small number of subjects included in this study is one of our limitations.

### Genetic Characteristics

In our series, the identification of EAS-related pathogenic variants further confirmed its genetic predisposition. So far, EAS was believed to be a complex polygenic hereditary disorder, while multiple genes were found to be related to EAS, such as *GRIN2A, KCNQ2, KCNQ3, DEPDC5, PAX6, GABRG2, RbFOX1/RbFOX3, SRPX2, FOXP2, PRRT2*, and other genes (5–16, 33). Recently, several studies have confirmed that the *GRIN2A* gene is the most likely candidate for EAS. The *GRIN2A* gene is located in chromosome 16p13.2 and encodes the *GluN2A* subunit of the *N*-methyl-d-aspartate receptor (NMDAR). NMDARs are hetero-tetramers usually composed of two GluN1 and two GluN2 (A-D) subunits and involved in neurodevelopment and brain plasticity, as well as learning, memory, and cognitive processes. NMDARs are activated following the binding of glycine and glutamate to the GluN1 and GluN2 subunits, respectively. GluN2 (A-D) subunit mainly consist of four domains: the extracellular amino (N)-terminal domain (NTD) which is involved in subunit assembly and allosteric modulation; the agonist-binding domain (ABD) which is made up of two discontinuous segments (S1 + S2); the transmembrane domain (TMD) which is composed of three and a half transmembrane helices (TM1–4) and forms the channel pore; and the intracellular C-terminal domain (CTD) involved in receptor trafficking (1, 15).

So far, more than 80 *GRIN2A* gene variants have been found in epilepsies, especially in EAS (1, 34). The more severe the phenotype is, the higher the positive rate of *GRIN2A* mutation is. It has been found that the positive rate of *GRIN2A* gene in the classical BECTS is 4.9%, in CSWs 17.6%, and in LKS 20% (7).

A recent study shows null variants of *GRIN2A* were associated with less severe EAS phenotypes and NMDAR-LOF (15). This finding is consistent with our outcomes: two ABPE (II) patients carrying *GRIN2A* null variants presented less severe DD/ID, despite the ESES-EEG existence. This finding further enforced that the severity of DD/ID might not parallel the severity of SWI, but genotype variants. However, due to the expensive testing fees, another limitation of this study is that only for eight cases whole-exome sequencing was available.

*De novo GRIN2A* variant c.2041C>T (p.Arg 681^*^) identified in our study was found to cause LKS (inherited) in 2013 (7). However, the symptoms of the patient in the previous study (manifesting no seizures, but with learning disability, auditory agnosia and receptive/expressive language disorder) were partially different from ours with linguistic impairments in common. The explanation could be the different inheritance pattern and phenotypic heterogeneity, which is worth further investigation.

As shown in Figure 3 and Table 7, EAS-related genes are enriched in the biological process of chemical synaptic transmission and vocalization (FDR, <0.01). As the key protein in PPI network, GluN2a protein coded by *GRIN2A* is linked to Foxp2-SRPX2 and the Pax6 protein through Rbfox3. The signal pathway of foxp2-srpx2/uPAR network is proved to be related to language disorders (35). The foxp2 protein is also regulated by the Pax6 protein. Therefore, we speculated that the *GRIN2A* gene might contribute to the language disorder of EAS through the foxp2-srpx2/uPAR pathway which is worth studying further.

### Treatment

Among the 17 patients treated with antiepileptic drugs, 12.5% were treated with monotherapy, 18.8% with multiple AED therapy, and 68.7% with multiple AEDs combined with steroids. Compared with the study of classic BECT children in Liu's group, where single drug treatment accounted for 62.9%, and multiple therapies accounted for 10.6% (26). Among the 11 children who received AEDs combined with steroid therapy, 72.7% of them had a good response (seizures reduction + seizure control), including one with the c.2094T>G (p.Y698^*^) variant. Our data suggested that the effect of monotherapy was not sufficient for severe subtypes of EAS or atypical BECTs, and most patients needed further methylpyridine pulse therapy after adding multiple AEDs. This was in concordance with current studies (36, 37). In 2015, a meta-analysis of 575 patients with ESES showed that corticosteroids and surgical treatment were the most effective way to reduce seizures and epileptic discharge and improve cognition, with an effective rate of 81 and 90%, respectively (36). A large-scale study of 44 patients with ESES also indicated that steroid therapy had a significant effect on the improvement of EEG and clinical neuropsychology of patients with ESES (38).

Notably, one patient with EEG-ESES carrying *GRIN2A* truncation variants which led to NMADR-LOF change our study reached cognitive recovery and SWI normalization ultimately by steroid treatment after given unresponsive AEDs. The mechanism could be as follows: (1) Autoimmune response was proved to contribute partially to the etiology of EAS. *GRIN2A* mutation itself could increase the antigenicity of extracellular components of NMDA receptor (22, 24, 39), and the seizure process might also increase the self-antigenicity of NMDA receptors (22). (2) Most likely, recent research presented that steroids can enhance the function of NMDA receptors and regulate glutamate activity. In 2012, Cynthia et al. found that hormones can increase the NMDA receptor numbers in 5-hydroxytryptamine neurons and enhance the activity of glutamate (40). In 2016, Swanger also found that pregnenolone sulfate can enhance NMDA function in LOF mutation of *GRIN2A in vitro* (20). Similar to our study, the previously reported patient with LKS who were treated with prednisone and immunoglobin after given unresponsive multiple AEDs, also noted significant improvement (22).

Therefore, for children carrying the *GRIN2A* mutation resulting in LOF of NMDAR, researchers may need to study whether neurologists should consider providing steroid treatment in time after given unresponsive AEDs, despite the absence of ESES signal on EEG to ensure a better prognosis.

For patients with pathogenic *GRIN2A* variants leading to NMDAR-GOF change, there are studies which argued that they mostly result in epileptic encephalopathy with more severe ID/DD and have poor prognosis (15). And the situation of these patients could be significant improved by memantine, a non-competitive antagonist of the NMDAR (41, 42). Those case reports guarantee the importance of precise treatment (19, 21, 23, 41, 42).

For now, the principle of treatment for EAS is not only to control the seizures but also to effectively decrease the SWI and improve the cognitive and linguistic outcomes (36). Earlier and appropriate intervention is always the priority to guarantee a good prognosis. Therefore, it is necessary to combine the epileptic syndromes, seizure types, neuropsychological status, SWI, and genetic characteristics of patients all together to conduct a comprehensive plan for the selection of AEDs. The effects of AEDs alone on cognitive improvement and seizure control are always insufficient for patients with EAS. So, methylpyridines pulse therapy should be given in a timely manner and monitored carefully due to the high relapse rate. However, under what certain circumstance should steroid be added still remains unclear in EAS. Especially for patients carrying *GRIN2A*-LOF variants present no ESES signal.

## Conclusions

In this study, we retrospectively reviewed the electroclinical characteristics and gene test results of children diagnosed with ABPE, LKS, and CSWS. Then, we further explored the GO of EAS-related genes and the PPI of those genes expressed. Our data suggested that when children suspected with BECTs have early-onset age, changed seizure semiology, and deterioration of behavior/cognition/motor function, neurologists should be alert on the appearance of ESES. The neuropsychological deterioration in children with EAS might not only be completely affected by electric discharge severity but also genetic etiology. Our finding also enforced the current genotype-phenotype relationship theory about EAS. For EAS children, GRIN2A-FOXP2-SRPX2/uPAR signal network might contribute to the mechanism of their language deficit.

## Data Availability Statement

The authors acknowledge that the data presented in this study must be deposited and made publicly available in an acceptable repository, prior to publication. Frontiers cannot accept a article that does not adhere to our open data policies.

## Ethics Statement

The studies involving human participants were reviewed and approved by the ethics committee of Children's Hospital of Chongqing Medical University. Written informed consent to participate in this study was provided by the participants' legal guardian/next of kin.

## Author Contributions

XL and LJ contributed to coming up with the ideas and data collection. XL and L-LX participated in co-writing. WH, S-QH, J-NM, and JW participated in the critical review of the manuscript. All authors have approved the manuscript for publication.

## Conflict of Interest

The authors declare that the research was conducted in the absence of any commercial or financial relationships that could be construed as a potential conflict of interest.
